# Supplementary material for: Structure of MlaFB uncovers novel mechanisms of ABC transporter regulation
Source: eLife. 2020 Jun 30;9:e60030. doi: 10.7554/eLife.60030 (PMC7367683; doi:10.7554/eLife.60030)
Supplement: Supplementary file 1. [file elife-60030-supp1.docx]

**Supplementary file 1: Bacterial strains.**

| **Strain** | **Relevant Genotype** | **Source** |
| --- | --- | --- |
| **MG1655** | F- lambda- ilvG- rfb-50 rph-1 | Blattner, F. R. et al. |
| **BW25113** | F-, Lambda-, ∆(araD-araB)567, lacZ4787(del)::rrnB-3, rph-1, ∆(rhaD-rhaB)568, hsdR514 | Datsenko, K. A. & Wanner, B. L. |
| **bBEL185** | *mlaB::gfp-FRT* | This work |
| **bBEL190** | *mlaF::FRT* | This work |
