# Supplementary material for: Structure of MlaFB uncovers novel mechanisms of ABC transporter regulation
Source: eLife. 2020 Jun 30;9:e60030. doi: 10.7554/eLife.60030 (PMC7367683; doi:10.7554/eLife.60030)
Supplement: Supplementary file 2. [file elife-60030-supp2.docx]

**Supplementary file 2: Plasmids.**

| **Plasmid** | **Relevant Features** | **Addgene ID** | **RRID** | **Source** |
| --- | --- | --- | --- | --- |
| **pBEL1200** | MlaFE-(6xHis-TEV-MlaD)-MlaCB | 155137 | Addgene_155137 | Ekiert, *et al*. |
| **pBEL1244** | MlaFE-(6xHis-TEV-MlaD)-MlaC | 155138 | Addgene_155138 | This work |
| **pBEL1245** | 6xHis-TEV-MlaF | 155139 | Addgene_155139 | This work |
| **pBEL1246** | 6xHis-TEV-MlaB | 155141 | Addgene_155141 | This work |
| **pBEL1266** | mlaF, tagless, araBAD promoter | 155142 | Addgene_155142 | This work |
| **pBEL1305** | (6xHis-TEV-MlaF)-MlaB | 155143 | Addgene_155143 | This work |
| **pBEL1306** | (MlaF-6xHis)-MlaB | 155144 | Addgene_155144 | This work |
| **pBEL1307** | MlaF-(6xHis-TEV-MlaB) | 155145 | Addgene_155145 | This work |
| **pBEL1308** | MlaF-(MlaB-6xHis) | 155146 | Addgene_155146 | This work |
| **pBEL1514** | mlaF(1-246)-mlaE-[6xHis-2xQH-TEV-mlaD]-mlaCB operon | 155147 | Addgene_155147 | This work |
| **pBEL1647** | mlaF(Δ247-267), tagless | 155148 | Addgene_155148 | This work |
| **pBEL1833** | mlaF(1-250)-GCN4 | 155149 | Addgene_155149 | This work |
| **pBEL1840** | MlaF-6xHis | 155150 | Addgene_155150 | This work |
| **pBEL1957** | Strep-MlaF-(6xHis-TEV-MlaB) | 155151 | Addgene_155151 | This work |
| **pBEL1965** | mlaF(1-246)-(6 aa linker)-GCN4 | 155152 | Addgene_155152 | This work |
| **pBEL1966** | mlaF(1-246)-(10 aa linker)-GCN4 | 155153 | Addgene_155153 | This work |
| **pBEL1967** | mlaF(1-246)-(14 aa linker)-GCN4 | 155154 | Addgene_155154 | This work |
| **pBEL2074** | Strep-MlaF-(6xHis-TEV-MlaB_T52E) | 155155 | Addgene_155155 | This work |
| **pBEL2076** | (Strep-MlaF_K47A)-(6xHis-TEV-MlaB) | 155156 | Addgene_155156 | This work |
| **pBEL2077** | (Strep-MlaF_E170Q)-(6xHis-TEV-MlaB) | 155157 | Addgene_155157 | This work |
| **pBEL2078** | (Strep-MlaF_Y261A)-(6xHis-TEV-MlaB) | 155158 | Addgene_155158 | This work |
| **pBEL2079** | (Strep-MlaF_L265A)-(6xHis-TEV-MlaB) | 155159 | Addgene_155159 | This work |
| **pBEL2080** | (Strep-MlaF_L266A)-(6xHis-TEV-MlaB) | 155160 | Addgene_155160 | This work |
| **pBEL2081** | (Strep-MlaF_Y261A_L265A_L266A)-(6xHis-TEV-MlaB) | 155161 | Addgene_155161 | This work |
| **pBEL2082** | (Strep-MlaFΔ[247-269])-(6xHis-TEV-MlaB) | 155162 | Addgene_155162 | This work |
