# Supplementary material for: Structure of MlaFB uncovers novel mechanisms of ABC transporter regulation
Source: eLife. 2020 Jun 30;9:e60030. doi: 10.7554/eLife.60030 (PMC7367683; doi:10.7554/eLife.60030)
Supplement: Supplementary file 3. [file elife-60030-supp3.docx]

**Supplementary file 3: Data collection and refinement statistics for crystal structures.**

**MlaF_2_B_2_ (ADP+Mg) MlaF_1_B_1_ (apo)**

**Data collection**

Space group: P3_2_21 P2_1_2_1_2_1_

Cell dimensions:

a, b, c (Å): 102.71 102.71 89.71 78.27, 136.08, 261.83

ɑ, β, ɣ (°): 90, 90, 120 90, 90, 90

Resolution (Å): 44.57-2.9 (3.004-2.9)^1^ 48.87-2.6 (2.693-2.6)^1^

Wavelength (Å): 1.11583 1.11583

Observations: 245,847 1,139,116

Unique Reflections: 12,466 86,874

Redundancy: 19.7 (20.3)^1^ 13.1 (12.6)^1^

Completeness (%): 93.09 (76.44)^1^ 92.07 (75.14)^1^

CC1/2: 0.999 (0.349)^1^ 1.00 (0.538)^1^

CC*: 1.00 (0.72)^1^ 1.00 (0.836)^1^

*I/σI* 16.09 (1.00)^1^ 18.68 (1.08)^1^

*R_meas_* 0.1835 (3.526) ^1^ 0.09513 (2.898)^1^

**Refinement**

Resolution (Å): 44.57 - 2.9 48.87-2.60

Reflections (work): 11,611 80,031

Reflections (free): 586 1,834

R_work_ / R_free_ (%): 19.42 / 24.95 20.28 / 23.65

No. atoms:

Protein: 2,760 11,361

Water: 2 10

Other: 29 62

Mean B-factor:

Protein: 82.75 92.2

Water: 42.31 65.04

R.M.S. Deviations:

Bond lengths (Å): 0.004 0.004

Bond angles (°): 0.79 0.81

Ramachandran plot:

Favored: 95.53% 99.86%

Outliers: 0.84 % 0.00%

Rotamer outliers: 0.00 % 0.65%

Molprobity:

Molprobity score: 1.58 1.44

Percentile: 100^th^ 100^th^

All-atom clashscore: 5.6 8.13

Percentile: 100^th^ 99^th^

PDB ID: 6XGY 6XGZ

^1^ Values in parentheses are for highest-resolution shell.
